# Revising the TIGER 2018 Health Informatics Competency Framework: Updated Core Competency Areas for Nurses in a Digital Health Era

**DOI:** 10.1055/a-2927-7621

**Published:** 2026-08-26

**Authors:** Ursula H. Hübner, Nicole Egbert, Kaija Saranto, Polun Chang, Ann Kristin Rotegård, Inge Cort Madsen, Zulma González, Elske Ammenwerth, Jen Bichel-Findlay, Marion J. Ball

**Affiliations:** 1 School of Business Management and Social SciencesCentre for Medical, Social and Health Informatics (MESH), Osnabrück University of Applied SciencesOsnabrückNiedersachsenGermany; 2 Department of Health and Social ManagementUniversity of Eastern FinlandKuopioFinland; 3 Institute of Biomedical Informatics, National Yang Ming Chiao Tung UniversityTaipeiTaiwan; 4 VAR HealthcareOsloNorway; 5 Department of Electrical and Computer EngineeringAarhus UniversitetAarhusCentral Denmark RegionDenmark; 6 Department of Health InformaticsUniversity Institute of the Italian Hospital of Buenos AiresBuenos AiresArgentina; 7 Institute of Medical Informatics, UMIT TIROL Private University for Health Sciences and Technology GmbHHall in TirolTyrolAustria; 8 Faculty of HealthUniversity of Technology SydneySydneyNew South WalesAustralia; 9 Department of Nursing/BioengineeringCenter for Innovation in Health Informatics (CIHI), The University of Texas at ArlingtonArlingtonTexasUnited States

**Keywords:** health informatics, nursing, education, recommendations, case studies

## Abstract

**Background:**

In 2018, the “TIGER International Recommendation Framework of Core Competencies in Health Informatics for Nurses” was published and used worldwide as a global guidance for educators and students to navigate educational requirements in an increasing digital working environment.

**Objective:**

Recent developments in digital health point to the need to revise these recommendations.

**Methods:**

This update followed the three-step logic of the 2018 framework development process, comprising (1) a global survey capturing the relevance of 43 core competency areas based on the ratings of 117 experts from all continents, (2) four regional expert workshops, and (3) 21 educational case studies. To ensure comparability with the 2018 framework, the same role-based structure was retained, covering Clinical Nursing, Nursing Management, Quality Management in Nursing, Coordination of Interprofessional Care, and Information Technology IT-Management in Nursing.

**Results:**

These recommendations underpin the high relevance level of health informatics for all nursing roles, characterizing each role by an informatics pattern: in Clinical Nursing patient issues, personal skills of working properly in a digital environment and a thorough understanding of the legal and ethical background of one's own work dominate the pattern. In Nursing Management, a similar composition prevails extended by management knowledge. The skill mix for Quality Management emerged as a blend of Clinical Nursing and Nursing Management, whereas Coordination of Interprofessional Care strongly emphasized coordination and teamwork capacities. IT Management in Nursing required basic and specialized computer skills incorporating problem solving, communication, and teamwork. These recommendations strongly suggest artificial intelligence (AI) topics should be addressed in all domains and for all roles of nurses.

**Conclusion:**

This updated recommendation framework provides guidance for focusing on areas that gained further priority such as patient safety, electronic health records, making use of its data and AI while retaining a core of invariant components of health informatics education for nurses.

## Introduction

In 2018, the “TIGER International Recommendation Framework of Core Competencies in Health Informatics for Nurses” was published as a global guidance for educators and students to navigate educational requirements in an increasing digital working environment. The work had been performed within two large projects in the United States& — funded by the Health Information and Management Systems Society (HIMSS) and Germany funded by the Federal Ministry of Education and Research. It was associated with the TIGER Initiative, founded in 2006 to boost education in digitally enabling technologies for nurses. 1 In recent years, the 2018 recommendation framework experienced worldwide recognition and application. 2 3 4 5 6 7 8 9 10 11 12 13 14 15 16 17 18 19 20 21

While this framework mainly addressed people seeking advice and orientation, it also offered and applied a methodology for developing and updating recommendations. 22 This approach embraced:

1. A quantitative priority rating of core competency areas in health informatics conducted via an online survey including global experts. The questionnaire underlying this survey had been constructed based on the current international literature to identify core competency areas.
2. A validation process of the questionnaire and the survey findings in regional expert workshops.
3. Local case studies from different countries to illustrate how these core competency areas were integrated into curricula, and practical teaching and learning situations of courses and entire study programmes. 22

According to the logic of these three steps the recommendations were tailored to five roles nurses can hold:

1. Clinical Nursing, i.e., the nurses at the point of care.
2. Nursing Management, i.e., nursing leadership.
3. Quality Management in Nursing, i.e., quality assurance and development in general and nursing in particular.
4. Information Technology (IT) Management in Nursing, i.e., working as a nurse informatics officer or similar roles that serve as an interface between the users and IT administration and development.
5. Coordination of Interprofessional Care, i.e., working as a case manager within an organization and across organizations and settings. 1 22

Given the goal that a digital working environment should streamline processes along the patient journey, professional or departmental silos should be avoided, and a broader perspective should be fostered. Pursuing this understanding renders nursing informatics firmly within health informatics without abandoning the special needs of nurses, e.g., regarding terminologies, work processes, and tasks. 23 24 25

By core competency areas, we understand generally accepted topics validated by the literature that are associated with cognitive competencies to achieve meaningful results. 1 Focusing on these areas, the 2018 Recommendations did not refer to individual competencies and competence levels as they strongly vary with the background of the target audience. Rather, they left it to the discretion of the educators themselves to decide on the need to briefly touch on a subject or to delve into the depth.

The 2018 Recommendation Framework, which included work from 2015 to 2017, was very much informed by the understanding of eHealth in a pre-coronavirus disease and pregenerative artificial intelligence (AI) era. Digitalization had been shaped by the possibilities of the technology to transform the paper forms into an electronic world — sometimes referred to as digitization. Thus, tools were used in a way that very much resembled the paper processes. 26 Changing the mindset from digitization to digitalization, opened up ample space for shaping new processes that capitalize on the typical features of digital media along the milestones of digitalization: from enabling advanced connectivity of persons and machines, mobility of information, to cognitive support of persons. 25 As health IT diffusion and adoption over the last few years resulted in a prolific digital environment in several areas of the world, 27 28 29 digital health as the product of health informatics has moved to the center of the debate. Digital health seeks to direct the gaze toward the health outcomes of digitalization, e.g., more effective, safe, and efficient health care services, improved outcomes for patients, and the public using and incorporating new technologies, 30 e.g., AI and human–technology teaming and new perspectives, e.g., risk management, governance, leadership, and innovation and implementation science. 31 However, “[t]here is no digital health without health informatics professionals able to select, design, implement, and deploy ICT resources to achieve digital health goals.” 32

The path from digitization to digital health is further illustrated by the latest trends of incorporating evidence into the health care and nursing processes in a more robust manner, 33 learning from one's own data in Learning Health Systems, 34 35 advanced clinical decision support, 36 37 and smart processes to enhance patient safety. 38 Further applications support the otherwise burdensome tasks of documentation. 39 Many of these use cases have recently been enhanced through AI methods such as prediction and classification algorithms, large language models, and generative algorithms, all of which depending on large amounts of high-quality data and information from health care including nursing.

With the advent of AI-enabled applications and their potential to revolutionize processes and practices, ethical and legal issues are coming to the fore that include patient privacy and data protection, trust, responsibility, accountability, liability, patient–provider interaction, and new biases introduced via the technology. 40

These developments point to the need for adjusting health informatics education to integrate knowledge and skills in AI, data science, and ethical and legal requirements. At the same time, health informatics education for nurses must be embedded in the current practice to address the daily challenges and enable nurses to master the basics of the technology.

## Objective

The overall goal, therefore, is to revise the 2018 findings regarding new competencies and an overall appraisal of high priority competency areas, while embracing the same methodological approach. This included the following questions that guided the revision: (1) What are the new competence area priorities of health informatics for the five nursing roles? (2) What are the changes between 2018 and today? (3) Are there priority differences regarding global regions? and (4) How can case studies illustrate and reflect different levels of education and training of nurses in a digital working environment?

## Methods

The update followed the three-step logic of the 2018 TIGER framework development process, comprising (1) a global survey, (2) regional expert workshops, and (3) educational case studies. 22 Fig. 1 shows the sequencing of the three steps and their main purpose. To ensure comparability with the 2018 framework, the same role-based structure was retained, covering five nursing roles.

**Fig. 1:**
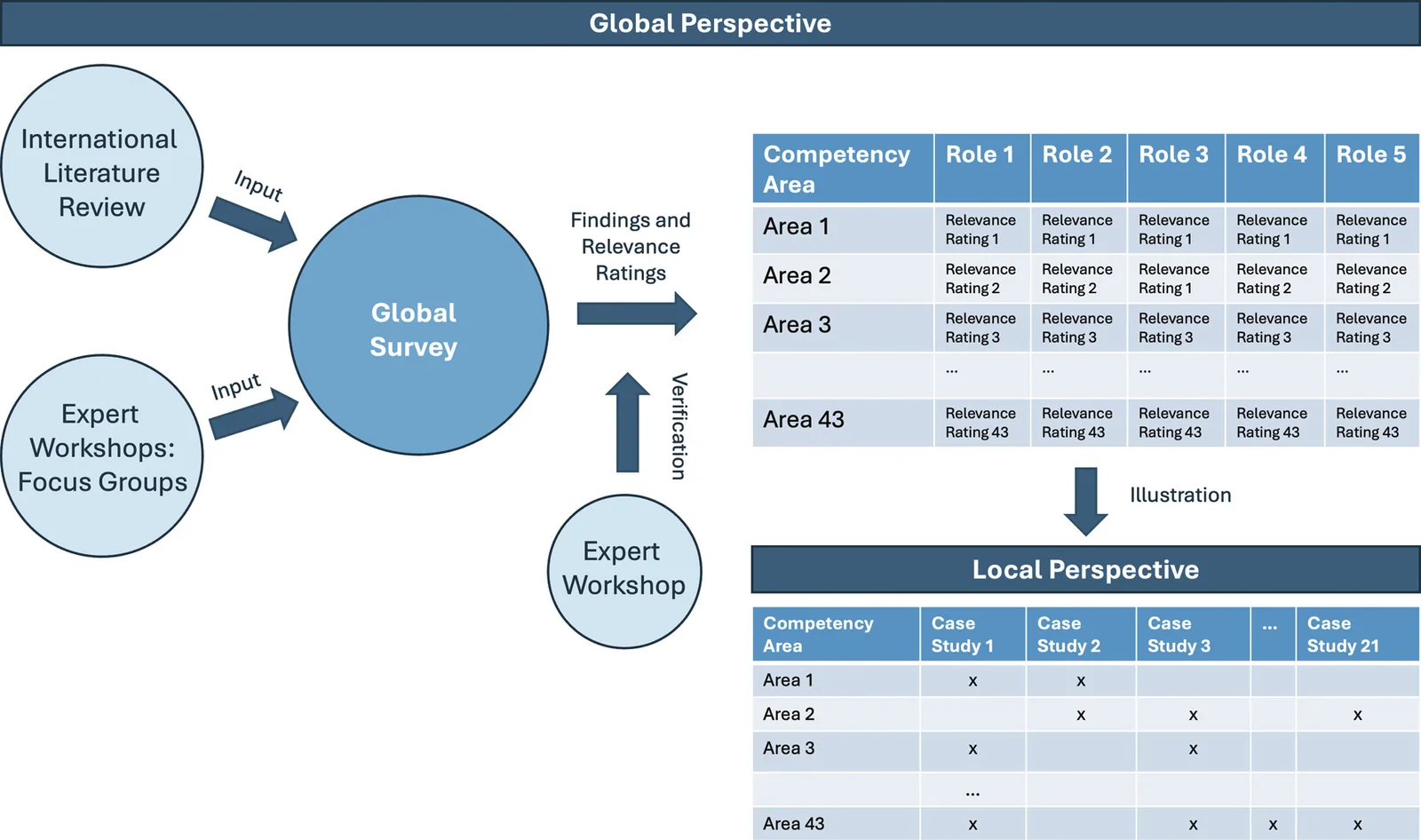
Three step methodology composed of survey, expert workshops, and case studies.

To identify competency areas that have become relevant for digitally supported nursing care since the original survey, a literature search was conducted in PubMed in May 2024, covering publications from 2019 onwards (search strategy provided in Supplementary Appendix A available in the online version only). This search identified 49 records. Additional records were identified through backward citation searching, involving the screening of reference lists of publications retrieved from PubMed, and through a supplementary nonsystematic freehand search using relevant keywords. These supplementary searches yielded 64 additional records, resulting in a total of 113 publications, including original studies and reviews ( Fig. 2 ).

**Fig. 2:**
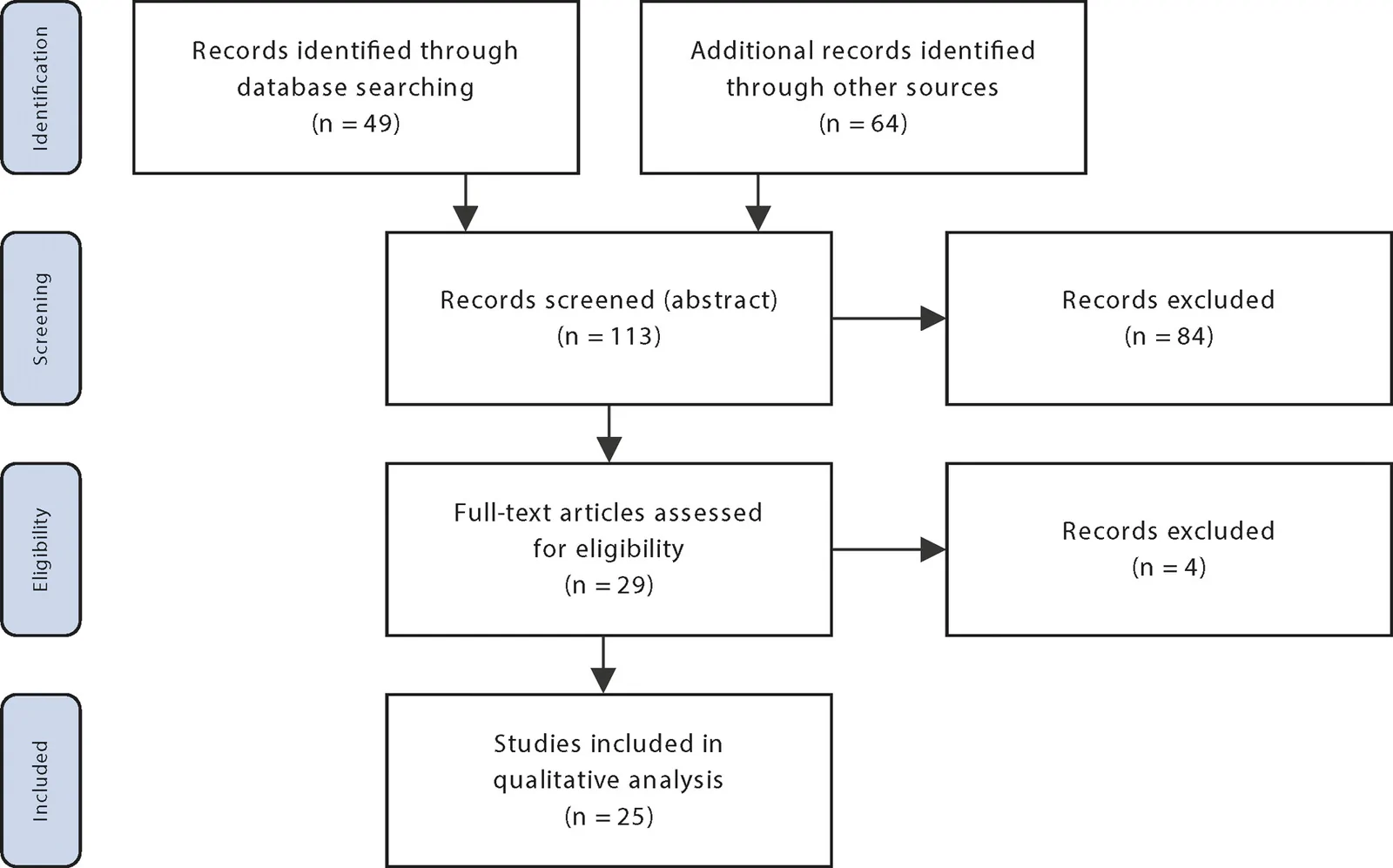
PRISMA flow diagram. PRISMA, Preferred Reporting Items for Systematic reviews and Meta-Analyses.

After title/abstract and full-text screening, 25 studies were included in the content analysis. Studies were excluded if competencies were not described in sufficient detail, competencies were not the main focus, the publication was methodological in nature (e.g., questionnaire development), or the study addressed veterinary or dental medicine. All records were screened for relevance by one reviewer (N.E.).

A data extraction table was used for content analysis. Extracted data included competency areas, competency descriptions, subcompetencies, and learning objectives where available. These findings were then systematically compared with the competency areas included in the original survey instrument to identify competency areas that remained unchanged, were newly added, or required adaptation.

Overall, 43 core competency areas were identified for the updated framework (see Supplementary Appendix B , available in the online version only). Of these, 18 remained unchanged, 16 were newly added ( Table 1 ), and 9 were renamed or adapted. The comparison was independently checked by a second reviewer (U.H.H.). The resulting set of competency areas formed the basis for the updated survey instrument.

**Table 1:** New competency areas 2026 compared with 2018

Number	New competency area 2026	Description/example
1	Artificial intelligence 42 43 44 45	Health-related AI applications, their capabilities and limitations; predictive AI techniques to improve the efficiency of patient care, ethical implications
2	Basic computer literacy/digital literacy 44 45 46 49 62 63 64 65 66 67 68 69 70 71	Basic word processing and spreadsheet, database, desktop presentation applications, web-based health information resources, internet resources, email, resolving minor technical problems
3	Care coordination and integration and health IT 42 45 49 68 70 72 73	Transition workflows within health care organizations and across different care settings, roles and responsibilities, process of transitioning patients between different levels and settings of care, strategies to ensure continuity of care
4	Clinical knowledge and health IT 20 42 44 45 46 63 73	Combining digital means and traditional methods, identify appropriate technology for patients, and adjust use of digital health as needed
5	Communication and health IT 42 66 67 70	Types and methods of communication, digital communication, verbal and nonverbal communication, sharing knowledge
6	Health equity and health IT 17 42 45	Social determinants for health, nonmedical factors influencing health outcomes, addressing specific needs and reducing disparities among diverse populations (e.g., cultural beliefs, language barriers)
7	Health care system, economics and regulations 17 42 45 46	Different types of national and international health care delivery models (e.g., hospitals, clinics, ambulatory centres and community health centres, regional health authorities) and their interrelationships
8	Innovation and creativity and health IT 42 44 70	Adoption and integration of innovative technologies, sharing innovative ideas, develop creative solutions; apply new ideas, products, processes, and procedures to individual work role or unit without fear of change
9	Interoperability 45 46 49 72	Health information standards and their appropriate use (e.g., classifications, vocabularies, nomenclature, etc.), information exchange between settings and health care providers
10	Patient education and engagement 42 45 63 65 70	Education and guidance of patients on self-management techniques, evaluate patient's digital capabilities
11	Patient safety 42 45 66 73	Safety protocols, guidelines, patient safety culture, reporting and learning from errors, incident reporting systems, monitoring patient outcomes
12	Problem solving and health IT 44 67 70	Dealing with digital problems, solving problems through digital means
13	Professional development and health IT 17 42 44 45 49 63 65 66 67 69 70	Lifelong learning, commitment to personal growth and self-development
14	Social and behavioral science and health IT 17 68 69	Effects of social, behavioral, and psychological models applicable to health informatics from multiple levels including individuals, organizations, social groups, and society
15	Sociotechnical aspects 17 45	Foundational knowledge, skills, and attitudes to identify social behavioral models and human factors engineering to better understand the interaction between users and information technologies and to be knowledgeable of information system design
16	Teamwork and collaboration and health IT 17 42 45 66 67 70 73	Tools such as online platforms for collaboration, document-sharing systems; roles/responsibilities for collaborative practice, interprofessional teamwork, and team-based practice

Abbreviations: AI, artificial intelligence; IT, information technology.

The educational approach reflected in these areas was further supported by recent research showing that 21 ^st^ century digital skills for nurses extend beyond technical abilities and are embedded in personal, social, and collective competence development. 41

The competency areas that had been investigated for the 2018 recommendations could be confirmed by the literature ( Supplementary Appendix B , available in the online version only). 42 43 44 45 46 47 48 49 50 51 52 53 54 55 56 57 58 59 60 61 62 63 64 65 66 All areas were mapped onto the domains of the International Medical Informatics Association's (IMIA) Biomedical and Health Informatics Education Recommendations of 2023. 25 Out of the 43 areas, 16 were assigned to Biomedical and Health Informatics (BMHI) Core Principles and Specializations, 4 to Health Sciences and Services (HS&S), 10 to Social and Behavioral Sciences (S&BS), 8 to Management Sciences (MS), and 5 to Computer, Data, and Information Sciences (CDIS). See assignment details in Supplementary Appendix C (available in the online version only).

The relevance of the individual competency areas was assessed using an online questionnaire created with LimeSurvey ( https://limesurvey.org ). Participants were asked to rate the relevance of all 43 core competency areas on a scale of 100% = very relevant to 0% not relevant at all. As in the 2018 survey, participants were asked to assess the relevance of the following five professional roles: Clinical Nursing (nurses at the point of care), Nursing Management (nurse leaders at all levels), Quality Management (nurses in charge of measuring and optimizing the quality of care and patient safety), Coordination of Interprofessional Care (e.g., nurses in interprofessional teams, discharge nurses, and nurses working at the intersection of professions and organizations), IT Management in Nursing (e.g., chief nurse informatics officers, nurse informaticians, nurse IT project, and process managers). A brief description was provided for each core competency area. Fig. 3 shows a short excerpt from the questionnaire as an example.

**Fig. 3:**
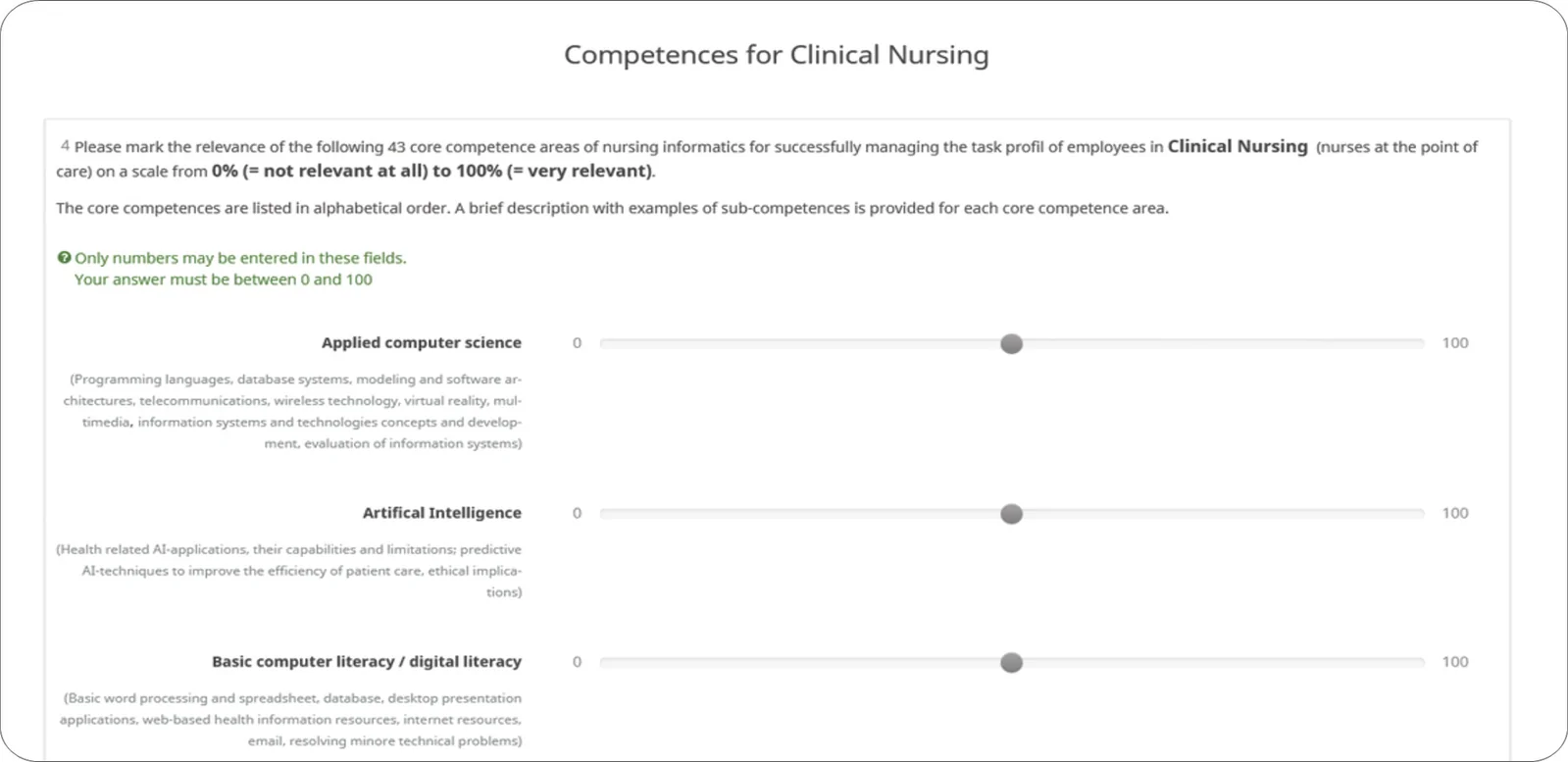
Competences for Clinical Nursing, relevance rating (example).

The invitation to participate in the survey was sent by email via various distribution lists to individuals working in the field of digitally supported care (including caregivers, scientists, and IT manufacturers) and distributed via other channels such as social media and conferences.

A total of 117 people from 28 different countries took part in the survey. Countries from every continent were represented: Europe (50.0%), North America (17.2%), Australia/Pacific (15.5%), Africa (8.6%), Asia (7.8%), and South America (0.9%) ( *n* = 116, one person did not provide country information).

When asked about their current professional practice, participants gave the following answers (number of responses in brackets): health care providers and education (33.3%), health informatics practice (29.1%), academia (28.2%), industry (3.4%), government and health authorities (2.6%), and other (3.4%). The professional background of the survey participants covered nursing (64.1%), other health sciences (26.5%), health informatics (0.9%), and other (8.5%).

### Workshops

In addition, expert consultations were conducted prior and after the survey. The pre-survey focus groups contributed to the refinement and extension of the competency areas identified through the literature search, whereas the post-survey workshop was used to discuss the survey findings and derive recommendations for the further development and application of the framework.

### Focus Groups ERASMUS (pre–survey)

The final list of core competency areas included the results of three focus group interviews with 21 health care experts that were conducted in Finland, Germany, and Portugal as part of the Erasmus+ Project eHealth4all@EU. The focus group interviews had shown that health informatics to support patient care, communication, ethics in health IT, change management, digital literacy, and information and knowledge management were broadly discussed and reflected crucial competency areas. 47

### Workshop at the Medical Informatics Europe (MIE) 2025 conference in Glasgow (post–survey)

In the postsurvey phase, a 1-hour workshop on “The Future of Nursing Informatics” was held at the MIE Conference in Glasgow, United Kingdom on May 9, 2025. The workshop agenda comprised an introduction, a presentation of the survey findings followed by a Question-and-Answer phase and a discussion. This workshop aimed to receive further experts' views on the survey findings.

### Case Studies

While the survey was meant to capture the global perspective, case studies were collected to reflect the local and practical perspective. Case studies were defined as descriptions of courses, study programmes, or other educational measures and their relation to implementing the core competency areas of the survey. To make the case study descriptions comparable, a template was developed from which those areas could be selected that were addressed by the educational case study. Further information could be provided regarding the academic or nonacademic level, whether it was a single course or a program, the course/program characteristics in terms of duration, credit points, the target group(s), the learning methods, the learning objectives, the assessment method and a short description of the content (see template sheet in Supplementary Appendix D , available in the online version only).

Case study authors were recruited at the international conferences IMIA Nursing Informatics NI 2024 in Manchester, UK and IMIA Medinfo 2025 in Taipei, Taiwan. In addition, selected persons from the nursing and health informatics field were approached personally or via IMIA officials. The goal was to garner diverse case studies from as many continents as possible. Representativeness of the case study for a region or an education type was not sought. Case studies were collected during the period from 2024 to 2026, most of them obtained in the first quarter of 2026.

## Results

### Priorities of Core Competency Areas

The priority lists for the core competency areas for health informatics in nursing within the recommendation framework are based on the relevance ratings of the international experts. Table 2 illustrates the top 10 competencies across the five roles. All top 10 core competency areas received relevance ratings exceeding 79% with means ranging from 83.4 to 87.9% ( Table 2 ). These values reflected generally high importance ratings for health informatics across the five roles. Although the mean relevance ratings did not differ substantially, the competency area profiles for the roles showed distinct patterns. For Clinical Nursing, competencies related to patient safety were rated the most relevant, followed by competencies in nursing documentation and basic computer literacy/digital literacy. In both the Nursing Management and IT Management in Nursing roles, basic computer literacy/digital literacy (lead) emerged as the most relevant competency domain. For Nursing Management, ethics and legal issues and principles of management ranked second and third, respectively, whereas for IT Management in Nursing, data protection and privacy and cybersecurity followed in the second and third place. Care coordination and integration and health IT was the leading competency area for the Coordination of Interprofessional Care role with basic computer literacy/digital literacy ranking third after ethics and legal issues. In the Quality Management role, quality assurance and quality management (lead), patient safety, and ethics and legal issues received the highest relevance ratings. Among the competency areas, basic computer literacy/digital literacy, and data protection and privacy stood out as they were represented among the top 10 in all five roles. All roles combined competency areas from all domains demonstrating that health informatics for nurses is composed of multidisciplinary elements and links BMHI Core Principles with Health Sciences and Services, Social and Behavioral Sciences, Management Sciences and Computer, Data and Information Sciences.

**Table 2:** Top 10 core competency areas in the five roles and related mean relevance (REL) (0 ... 100%)

Clinical Nursing, mean = 83.6 ± 19.8						Quality Management, mean = 86.0 ± 18.4					
Domain		Core competency area	REL ± SD	95% CI		Domain		Core competency area	REL ± SD, *n* = 69	95% CI
HS&S	1	Patient safety [ *n* = 112]	88.1 ± 17.6	84.9	91.4	MS	1	Quality assurance and quality management	91.5 ± 15.6	87.8	95.1
BMHI	2	Nursing documentation [ *n* = 112]	86.8 ± 18.0	83.5	90.1	HS&S	2	Patient safety	90.0 ± 15.6	86.3	93.7
BMHI	3	Basic computer literacy/digital literacy [ *n* = 117]	86.0 ± 20.6	82.2	89.7	S&BS	3	Ethics and legal issues	89.0 ± 15.6	85.3	92.7
S&BS	4	Ethics and legal issues [ *n* = 113]	83.9 ± 19.7	80.3	87.5	BMHI	4	Basic computer literacy/digital literacy	85.7 ± 19.8	81.0	90.3
S&BS	5	Data protection and privacy [ *n* = 113]	83.6 ± 20.7	79.8	87.4	BMHI	5	Data literacy	85.7 ± 20.2	80.9	90.4
HS&S	6	Patient education and engagement [ *n* = 112]	82.9 ± 20.3	79.1	86.6	S&BS	6	Data protection and privacy	85.7 ± 20.6	80.8	90.5
S&BS	7	Communication and health IT [ *n* = 113]	82.9 ± 20.0	79.1	86.5	CDIS	7	Data analytics	83.9 ± 18.0	79.7	88.2
BMHI	8	Clinical knowledge and health IT [ *n* = 113]	81.7 ± 19.2	78.1	85.2	BMHI	8	Nursing documentation	83.6 ± 19.6	79.0	88.2
BMHI	9	eHealth and electronic health records [ *n* = 113]	80.2 ± 20.9	76.3	84.0	BMHI	9	Information and knowledge management in patient care	82.9 ± 18.4	78.6	87.2
BMHI	10	Care coordination and integration and health IT [ *n* = 115]	79.8 ± 20.9	76.0	83.6	MS	10	Process management	82.0 ± 19.4	77.5	86.6
**Nursing Management, mean = 87.1 ± 18.2**						**IT Management in Nursing, mean = 87.9 ± 18.7**					
Domain		Core competency area	REL ± SD. *n* = 81	95% CI		Domain			Core competency area	REL ± SD. *n* = 64	95% CI
BMHI	1	Basic computer literacy/digital literacy	89.3 ± 16.9	85.5	92.9	BMHI	1	Basic computer literacy/digital literacy	90.6 ± 19.6	85.8	95.4
S&BS	2	Ethics and legal issues	88.9 ± 16.9	85.2	92.6	S&BS	2	Data protection and privacy	89.5 ± 17.9	85.1	93.9
MS	3	Principles of management	88.5 ± 17.3	84.7	92.3	CDIS	3	Cybersecurity	89.2 ± 17.6	84.9	93.5
S&BS	4	Data protection and privacy	88.2 ± 21.2	83.5	92.8	BMHI	4	eHealth and electronic health records	88.9 ± 18.2	84.5	93.4
HS&S	5	Patient safety	87.8 ± 18.0	83.8	91.7	BMHI	5	Principles of nursing informatics	88.0 ± 18.5	83.4	92.5
BMHI	6	Care coordination and integration and health IT	87.0 ± 16.0	83.6	90.5	S&BS	6	Problem solving and health IT	87.8 ± 19.1	83.1	92.5
BMHI	7	Nursing documentation	85.7 ± 18.3	81.7	89.7	S&BS	7	Communication and health IT	87.7 ± 15.7	83.8	91.5
S&BS	8	Change and stakeholder management	85.4 ± 19.1	81.3	89.6	CDIS	8	IT risk management	86.7 ± 19.4	82.0	91.5
MS	9	Strategic management. Leadership and intrapreneurship	84.9 ± 20.3	80.5	89.4	S&BS	9	Teamwork and collaboration and health IT	85.3 ± 19.9	80.4	90.2
MS	10	Human resource management	84.8 ± 17.8	81.0	88.7	BMHI	10	Data literacy	85.2 ± 20.4	80.2	90.2
**Coordination of Inter-professional Care mean = 83.4 ± 19.5**											
Domain		Core competency area	REL ± SD	95% CI							
BMHI	1	Care coordination and integration & health IT [ *n* = 62]	87.6 ± 18.4	83.0	92.2						
S&BS	2	Ethics and legal issues [ *n* = 62]	86.0 ± 19.5	81.1	90.8						
BMHI	3	Basic computer literacy / digital literacy [ *n* = 62]	84.8 ± 19.4	80.0	89.7						
HS&S	4	Patient safety [ *n* = 62]	84.7 ± 17.5	80.3	89.0						
S&BS	5	Teamwork and collaboration & health IT [ *n* = 62]	83.6 ± 20.3	78.5	88.6						
S&BS	6	Communication & health IT [ *n* = 63]	83.3 ± 19.0	78.6	88.0						
BMHI	7	Nursing documentation [ *n* = 62]	82.4 ± 19.0	77.7	87.1						
S&BS	8	Data protection and privacy [ *n* = 61]	81.6 ± 23.4	75.8	87.5						
BMHI	9	Information and knowledge management in patient care [ *n* = 62]	80.3 ± 18.6	75.7	84.9						
BMHI	10	Information and communication systems for nursing [ *n* = 62]	79.5 ± 19.9	74.6	84.5						

Abbreviations: BMHI, Biomedical and Health Informatics Core Principles; CDIS, Computer, Data and Information Science; CI, confidence interval; HS&S, Health Sciences and Services; MS, Management Science; REL, relevance; S&BS, Social and Behavioral Sciences; SD, standard deviation.

To identify the depth of health informatics needs per role, the number of core competence areas with an average rating of at least 80% were counted. The analysis revealed that IT Management in Nursing comprised the largest number (26). Nursing Management followed with 23 core competency areas, whereas Quality Management (11), Coordination of Interprofessional Care (9), and Clinical Nursing (9) exhibited only half or less of this number. See Supplementary Appendix E (available in the online version only) for all ratings.

### Clinical Nursing in Global North versus Global South

While the core competency areas in the list of the global north ( *n* = 98 experts) were identical to the overall list ( Table 2 ), there were two differences compared with those from the global south ( *n* = 18). Instead of selecting clinical knowledge and health IT, and care coordination and integration and health IT, experts from the global south voted for principles of nursing informatics and information and knowledge management in patient care.

### Changes of Priorities from 2018 to 2026

Changes to the 2018 high priority core competency areas are shown in Table 3 for the roles Clinical Nursing, Nursing Management, and IT Management in Nursing. eHealth and electronic health records as well as data analytics experienced the largest increases in the respective roles with more than 10 points higher in all but one instance. All changes can be found in Supplementary Appendix F (available in the online version only).

**Table 3:** Changes in relevance ratings from 2018 to 2026 for the roles Clinical Nursing, Nursing Management, IT Management in Nursing from 5 points (measured in %) minimum; areas in bold mark differences of more than 10 points

Clinical Nursing				Nursing Management			
**Competence area**	**2026**	**2018**	**Difference**	**Competence area**	**2026**	**2018**	**Difference**
At least 5 points higher relevance rating in 2026 compared with 2018				At least 5 points higher relevance rating in 2026 compared with 2018			
eHealth and electronic health records	80.2%	69.5%	10.7%	Ethics and legal issues	88.7%	80.5%	8.2%
Decision support and decision-making	76.6%	70.2%	6.4%	Data protection and privacy	87.6%	80.2%	7.4%
Data analytics	59.4%	47.8%	11.6%	Resource planning and logistics	84.3%	76.0%	8.3%
Information management in research	57.1%	51.0%	6.1%	eHealth and electronic health records	83.8%	66.3%	17.5%
At least 5 points lower relevance rating in 2026 compared with 2016				Project management	83.5%	76.3%	7.2%
Nursing documentation	86.8%	94.4%	−7.6%	Financial management	81.0%	73.7%	7.3%
Principles of nursing informatics	71.6%	80.5%	−8.9%	Information and communication systems for nursing	80.6%	75.1%	5.5%
Quality assurance and quality management	66.7%	72.0%	−5.3%	Decision support and decision-making	79.8%	74.7%	5.1%
Process management	58.6%	67.8%	−9.2%	Data analytics	79.2%	59.5%	19.7%
Principles of management	53.0%	59.8%	−6.8%	Information management in research	71.3%	63.3%	8.0%
Human resource management	49.9%	57.1%	−7.2%	At least 5 points lower relevance rating in 2026 compared with 2018			
Applied computer science	46.0%	53.7%	−7.7%	Nursing documentation	86.5%	92.1%	−5.6%
**IT Management in Nursing**							
**Competence Area**	**2026**	**2018**	**Difference**				
At least 5 points higher relevance rating in 2026 compared with 2018							
eHealth and electronic health records	88.9%	80.0%	8.9%				
Data analytics	81.1%	67.3%	13.8%				
At least 5 points lower relevance rating in 2026 compared with 2018							
Information and knowledge management in patient care	78.6%	86.1%	−7.5%				

Abbreviation: IT, Information Technology.

When comparing the core competency areas that did not change their listing among the top 10 over the years (even if their rating level had changed), Clinical Nursing was characterized by nursing documentation, ethics and legal issues, data protection and privacy, and eHealth and electronic health records. Nursing Management showed an overlap of competency areas for nursing documentation, ethics and legal issues, principles of management, strategic management and leadership, change management and stakeholder management, and human resource management. Finally, IT Management in Nursing shared the competency areas principles of nursing informatics, data protection and privacy, and IT risk management. These competency areas formed a set of invariants that established the core of health informatics for nurses over the years ( Supplementary Appendix F , available in the online version only).

### Workshop (post-survey)

The presentation of the survey findings at the MIE 2025 workshop was followed by a debate on whether AI was a single core competence area or whether it belonged to a whole group of areas. Most participants favored the latter understanding of AI. Similarly, the importance of competence areas such as 21 ^st^ century skills was reflected in the discussion. Stimulated by the high relevance ratings of basic computer literacy and digital literacy, the need was expressed to adapt the recommendations to the cultural and digital ecosystem when applied in a specific country. The participants shared the view that the findings from the survey were rather general and required the case studies to render them more tangible and practically usable.

### Case Studies

A total of 21 educational case studies ( Table 4 ) could be collected, of which seven originated from Asia, six from Europe, five from North America, and three from South America. The target audience was either interdisciplinary (7) or purely nursing-related (14). Among the latter specific competency patterns were addressed for specific roles (11), whereas a small group addressed all roles for nurses (3). Broken down by role this picture could be subdivided into Clinical Nursing (7), Nurses Management including Quality Management (2), IT Management in Nursing (1), and a combination of IT Management in Nursing and Nursing Management (1). Three of the interdisciplinary case studies included aspects of the role Coordination of Interprofessional Care. In summary, all roles were covered by at least one educational case study. The IMIA BMHI domains were either covered completely through specific core competency areas (six case studies) or combinations of domains were comprised, very often including the domains Biomedical and Health Informatics, Health Sciences and Services, and Social and Behavioral Sciences. The case study collection embraces the full spectrum of levels from vocational training, undergraduate, and postgraduate studies to further education.

**Table 4:** Case study overview

**Tokyo University Department of Nursing Data Science and Nursing Administration, Japan**						
	Role and target group	Level	Course type	Main domains according to the IMIA	Focus	Author(s)
Tokyo 1	Nursing Management	Research basics	Practicum	CDIS and BMHI	Data analytics	Haruna Kugai, Taisuke Yasaka, Kojiro Morita, Ai Tomotaki, Kenshi Hayashida, Mari Ikeda
Tokyo 2	Nursing Management and Quality Management	Research	Single course: in person	CDIS, BMHI, MS, and S&BS	Real-world data, data analytics	
Tokyo 3	Clinical Nursing and interdisciplinary and Care Coordination and Integration	Research for a broader audience	Online seminar	CDIS, BMHI, MS, and S&BS	Real-world data, data analytics	
Tokyo 4	All nursing roles	Research	Regular group meetings	All domains	Real-world data, data analytics	
**University of the Philippines, Medical Informatics Unit, Philippines**						
	Role and target group	Level	Course type	Main domains	Focus	Author(s)
Philipp 1	Interdisciplinary, primary care providers	Introduction for vocational training and further education	Single course: hybrid course	BMHI, HS&S, S&BS, and CDIS	Foundations of digital health	Fatima Sucaldito, Lisa Traboco,Iris Thiele Isip-Tan
**National Yang Ming University, Taipei, Taiwan, Taiwan Nursing Informatics Association and partners in China**						
	Role and target group	Level	Course type	Main domains	Focus	Author(s)
TNIA1	Clinical Nursing and other nursing roles	All levels	Series of courses: in person and online courses	All domains	Very comprehensive Nursing Informatics program	Polun Chang
**Samsung Medical Centre, Seoul, Korea**						
	Role and target group	Level	Course type	Main domains	Focus	Author(s)
Samsung 1	Clinical Nursing	Further education	Single course: in person and online course within hospital	Elements from all domains	Specialized program on AI-enabled decision-making and data literacy	Jeong Hee Hong
**NURIC consortium funded by ERASMUS with UMIT Tyrol/Austria as coordinator (** **https://nuric-eu.com/our-partners/** **), Austria, Israel, Italy, Kosovo**						
	Role and target group	Level	Course type	Main domains	Focus	Author(s)
NURIC 1	Clinical Nursing	Vocational training, Bachelor and further education	Various courses: in person and online course within hospitals and universities	BMHI, HS&S, S&BS, and CDIS	Basis TIGER 2018 Clinical Nursing core competency areas plus national demands, e.g., telehealth, for the digital transformation of health care	Elske Ammenwerth
**National and Kapodistrian University of Athens (Department of Nursing and Economics), University of Piraeus (Department of Informatics and Digital Systems, Greece**						
	Role and target group	Level	Course type	Main domains	Focus	Author(s)
Kapodi 1	Interdisciplinary	Postgraduate	Single course; hybrid	BMHI, HS&S, S&BS, and CDIS	Telemedicine including AI	Parisis Gallos
**University of West Attica Athens, Nursing Department, School of Health and Care Sciences, Greece**						
	Role and target group	Level	Course type	Main domains	Focus	Author(s)
Attica 1	Nursing in all roles	Undergraduate	Single course: in person	BMHI, HS&S, S&BS, and CDIS	Overview nursing informatics and applications	Parisis Gallos
**University of Eastern Finland, Master Programs in Health and Human Services Informatics, Health and Social Management, Rescue Services Management, Kuopio, Finland**						
	Role and target group	Level	Course type	Main domains	Focus	Author(s)
Kuopio 1	Interdisciplinary	Postgraduate	Single course; hybrid	Elements from S&BS and CDIS	Cybersecurity, ethics, IT risk management	Ulla Mai Kinnunen
**Health Campus Göttingen, Germany**						
	Role and target group	Level	Course type	Main domains	Focus	Author(s)
Gött 1	Interdisciplinary health care providers	Undergraduate	Single course; hybrid	BMHI, HS&S, S&BS, and CDIS	Basics of technology supported coordination of interprofessional care	Björn Sellemann
Gött 2	Interdisciplinary health care providers	Undergraduate	Single course; hybrid	BMHI, HS&S, S&BS, and MS	Technology supported coordination of interprofessional care	
**University Hospital Italiano Buenos Aires, Buenos Aires, Argentina**						
	Role and target group	Level	Course type	Main domains	Focus	Author(s)
HIBA 1	Clinical Nursing	Undergraduate (4th year)	Single course: online	BMHI, HS&S, S&BS	Fundamental concepts of health informatics for the digital transformation of health care	Zulma González
HIBA 2	Clinical Nursing (RN) working in health care organizations	Postgraduate further education	Single course: online	BMHI, HS&S, S&BS, and MS	Specialized course to obtain a deeper understanding of nursing informatics to enable EHRs, documentation, workflows, decision-making, patient safety	
**National University of José C. Paz Buenos Aires, Argentina**						
	Role and target group	Level	Course type	Main domains	Focus	Author(s)
Paz 1	Clinical Nursing	Undergraduate (2nd year)	Single course: hybrid	BMHI and CDIS	Fundamental concepts of computing and digital technologies, including hardware, software, and cloud-based tools with focus on information literacy	Zulma González
**Johns Hopkins University Baltimore, School of Nursing, United States**						
	Role and target group	Level	Course type	Main domains	Focus	Author(s)
Hopkins 1	Clinical Nursing	Postgraduate (1st clinical semester)	Single course: hybrid	BMHI, HS&S, and S&BS	Clinical judgement with the enabling technologies of evidence-based practice also relating to patient safety	Krysia Hudson
**University of Texas at Arlington, College of Nursing, United States**						
	Role and target group	Level	Course type	Main domains	Focus	Author(s)
UTA1	Clinical Nursing	Postgraduate	Single course; online	BMHI, HS&S, S&BS, and MS	Mobile app application for patient engagement	Mari Tietze
UTA2	IT Management in Nursing: advanced NI role	Postgraduate	Series of courses: hybrid	All domains	Nursing informatics specialty courses within Master of Nursing Sciences covering the full spectrum of the core competency areas	Karen Knecht
UTA3	IT Management in Nursing and Nursing Management: professional nurses: advanced practice informatics roles	Postgraduate	Single course: hybrid	Selected elements of all domains	Managing and leading in informatics health policy	
**Texas Woman's University, Denton, United States**						
	Role and target group	Level	Course type	Main domains	Focus	Author(s)
TWU 1	Interdisciplinary	Postgraduate	Entire program in Data Science and Community Informatics: online and in person	All domains	Integration of computer science, cognitive science, and organizational science in health care to address the complex relationship between information, technology and society focusing on data analytics	Erin Dickerman

Abbreviations: AI, artificial intelligence; BMHI, Biomedical and Health Informatics Core Principles; CDIS, Computer, Data and Information Science; EHR, electronic health record; HS&S, Health Sciences and Services; IMIA, International Medical Informatics Association; IT, information technology; MS, Management Science; S&BS, Social and Behavioral Sciences; TWU, Texas Woman's University; UTA, University of Texas at Arlington.

In most cases, a single course was described. However, there were also two case studies reporting on a series of courses and one that provided details on a full study program. Nearly all case studies referred to a hybrid course delivery mode.

The case studies are found in full length at https://netcase.hs-osnabrueck.de/index.php/s/UieYPnczizCTHfV .

## Discussion

### Summary

This revision of the Recommendation Framework of Core Competency Areas in Health Informatics for nurses aimed at providing the most recent recommendations rooting them in global empirical findings (survey and case studies) based on an updated list of core competency areas. To this end, the original list of 24 core competency areas was extended to 43 potential areas based on the literature review and the workshops prior to the survey. Among them, the top 10 cover the core competency area priority list for each role of the recommendation framework. The postsurvey workshop verified the methodological steps and added further recommendations. Compared with the 2018 version, the number of participants for the survey and of the case studies could be increased.

The 2026 findings underpin a high relevance of health informatics for different roles in nursing making no substantial distinction between the roles concerning the average level of relevance. This justifies and demands the inclusion of health informatics in all educational measures irrespective of the tasks and roles. However, each role is characterized by a custom pattern of core competency areas: in Clinical Nursing patient issues, personal skills of working properly in a digital environment and a thorough understanding of the legal and ethical background of one's own work dominate the pattern. In Nursing Management, a similar pattern prevails extended by management skills and knowledge. The skill mix for Quality Management emerges as a blend of Clinical Nursing and Nursing Management, whereas Coordination of Interprofessional Care strongly emphasizes coordination, cooperation, and teamwork capacities in addition. IT Management in Nursing requires basic and specialized computer skills incorporating problem solving, communication, and teamwork. Judged by the number of core competency areas with relevance ratings of 80% or more IT Management in Nursing and Nursing Management exhibited more core competency areas to be covered than the other roles speaking in favor of a track of courses or an entire study program in health informatics rather than a single course for these two roles. While it does not seem surprising that specialists in IT Management in Nursing require a comprehensive education in health informatics, the need for nurse leaders (Nursing Management) to possess extensive health informatics capacities seems unusual. However, the work of nurses is determined largely by the work processes of the organization, and it is the role of the leadership to foster digital innovation to the benefit of patients and the health care providers. 48

The case studies provide evidence of how such courses, tracks or series, and programmes can be put into practice either as interdisciplinary or nursing-specific educational measures, very often employing hybrid teaching and learning methods combining in-person and online elements. Many of the case studies aim at a larger bundle of core competency areas to show the full spectrum of what it entails to apply IT in patient care. Others go for selected topics, e.g., analysis of real-world databases, ethics, and data protection and privacy, AI-enabled decision-making, or telemedicine/telehealth.

In summary, these findings provide guidance for tailoring health informatics courses for nurses in different roles. These recommendations locate health informatics for nurses firmly within the multidisciplinary canon of IMIA's recommendations in Biomedical and Health Informatics education 45 and specify them for the specific needs of nurses. These recommendations mainly inform education but have also implications for practice and research regarding their interaction with education.

### Basic Computer Literacy/Digital Literacy

It may appear surprising that basic computer literacy and digital literacy still rank very high. One would expect that the generation of “digital natives” did not need dedicated training in this field. Obviously, this is not the case. Furthermore, basic computer literacy also covers many practical skills and hands-on knowledge identified as high-priority areas, 49 an area that constantly needs updates with tools changing over the years and incorporating basic AI literacy as well.

### Artificial Intelligence

With the advent of AI supported tools and applications intruding into the private sphere of patients, it is not surprising that ethics and/or data protection and privacy are among the top 10 for all roles. As AI applications have more slowly pervaded the work of nurses than of other health care providers, e.g., radiologists and dermatologists, skills, and knowledge touching AI as a single core competency area were not to be found among the top 10 and only in a few case studies as a topic of its own. However, the experts laid the cornerstone for using AI applications emphasizing the importance of data literacy, data analytics, ethics, data protection, and privacy among the top 10 core competency areas.

Supported by the post-survey workshop discussions, these recommendations strongly suggest AI topics should be addressed in all domains not only in computer, data, and information science and for all roles of nurses. This includes practical experience handling AI-enabled tools and applications as part of the education. 50 It further embraces generative AI literacy, 51 critical thinking, and output appraisal 52 fueled by methods to carry out validation studies for in-house developments and particularly for purchased applications to make sure the underlying models fit the local patient population(s), as had been demonstrated a couple of years ago in radiology. 53 It also encompasses a general understanding of the strengths and weaknesses of AI applications raising the awareness of potential biases that may influence personal decision-making behavior and the patient–provider relationship. 40 Last, but not least, these recommendations embrace methods to develop organizational governance rules for AI implementation and use based on the legal and ethical foundations and on implementation science knowledge. 54 Emerging from a discussion with nursing informatics leaders, strong leadership — as reflected by the core competency areas for nursing management in these recommendations — was portrayed as a pivotal element. Based on the Non-Adoption, Abandonment, Scale-up, Spread, Sustainability framework the nursing informatics leaders emphasized among others the value proposition of AI and the external conditions as key to AI adoption in nursing. 55 AI innovations will be particularly based on intrapreneurship of nurses taking responsibilities and risks for the organization to eventually spur the digital transformation of the patient journey.

These topics have to be adjusted based on the actual diffusion of AI in health care and its use cases and should account for general insights on innovation derived from Gartner's hype cycle 56 to guide strategic adoption in practice.

### Limitations

These recommendations are based on a sample that covers many countries around the globe including all continents; however, experts from Europe and North America constituted the largest group of experts. Furthermore, most of the experts in all phases of the development were handpicked based on their connection to the IMIA and the European Federation of Medical Informatics (EFMI) either through the working groups of interest, the member societies, the attendance of IMIA and EFMI conferences or other relations to the nursing informatics community (universities, health care organizations, industry etc.). Thus, their views reflect a broad perspective as the distribution of the survey participants across the different practice areas revealed. With over 100 expert voices in the survey heard, this recommendation is based on a larger survey sample than the 2018 recommendations 1 ; however, it was smaller than the sample used for TIGER recommendations covering a comprehensive set of different roles of health care professionals. 57 Although there were survey participants from Africa, no case study authors from African countries could be identified who were willing to provide information on their health informatics courses for nurses.

The professional composition of the sample should also be considered when interpreting the findings. While two thirds of the respondents had a professional background in nursing, one third came from other health sciences, health informatics, or other professional fields. Moreover, participants represented a broad range of current areas of practice, particularly health care provision and education, health informatics practice, and academia. This interdisciplinary composition is valuable because nursing informatics is situated within health informatics at the interface of nursing practice, education, research, and IT. Participants from related disciplines may therefore provide relevant perspectives on the competencies required for digitally supported nursing care, particularly in the role of coordinating interprofessional care. However, their assessments may differ from those of nurses and may have influenced the survey results.

Despite the need to adjust these recommendations to the cultural and local priorities, they provide guiding principles similarly valid for countries in the Global North as well as from the Global South as the comparison of the top 10 ratings for Clinical Nursing had demonstrated. As shown in a study on nursing informatics courses in Latin America and the Caribbean, 58 universities from these countries seek to align their course content with international recommendations.

These recommendations do not explicitly encompass the role of the health informatics teacher and researcher. The teachers' competency areas should align with the recommendations for the target audience of their courses, whereas the researchers usually identify the topics and tools through the exchange with the scientific community not via recommendations.

### Outlook

Intended to inform education, the recommendations also have the potential to impact research on high priority topics, e.g., AI and data ethics, 59 communication and health IT, 60 and practice via directing the focus to major applications and methods, e.g., patient safety and fall risk assessment. 61

## Conclusion

This updated recommendation framework provides guidance for focusing on areas that gained further priority such as patient safety, electronic health records, and making use of patient data as well as AI, while retaining a core of invariant components of health informatics education for nurses that comprise nursing documentation, ethical and legal issues, data protection, and privacy as well as various management skills, IT risk management, and principles of nursing informatics depending on the respective roles. The recommendations are tailored to the profile of different nursing roles showing that one size does not fit all. Due to the rapid changes in digital health, a regular revision of the recommendation framework for health informatics core competency areas for nurses seems advisable.
