# Supplementary material for: Revising the TIGER 2018 Health Informatics Competency Framework: Updated Core Competency Areas for Nurses in a Digital Health Era
Source: Methods Inf Med. 2026 Aug 26;65(01-02):13–27. doi: 10.1055/a-2927-7621 (PMC13668397; doi:10.1055/a-2927-7621)
Supplement: Supplementary file 1 [file 10-1055-a-2927-7621_29458027.pdf]

Appendix A

Search String

The PubMed search strategy was: (competenc*[Title/AbsStract]) AND (health informatic*[Title/Abstract]). Filters applied were free full text, publications from the last five years, English or German, and sorting by First Author.

Appendix B

Comparison of Core Competence Areas 2018 vs. 2026

No.	2018	2026	Description/Example	Study Reference
1	Applied computer science	Applied computer science	Programming languages, database systems, modeling and software architectures, telecommunications, wireless technology, virtual reality, multimedia, information systems and technologies concepts and development, evaluation of information systems	[20,44-46,47,64,67,72]
2		Artifical Intelligence	Health related AI-applications, their capabilities and limitations; predictive AI-techniques to improve the efficiency of patient care, ethical implications	[42-45]
3		Basic computer literacy / digital literacy	Basic word processing and spreadsheet, database, desktop presentation applications, web-based health information resources, internet resources, email, resolving minore technical problems	[44-46,49,62,71]
4		Care coordination and integration & health IT	transition workflows within healthcare organisations and across different care settings, roles and responsibilities, process of transitioning patients between different levels and settings of care, strategies to ensure continuity of care	[42,45,49,68,70,72,73]
5	Change and stakeholder management	Change and stakeholder management	Tasks and measures to bring a comprehensive change (new strategies, structures, processes or practices; taking into account all stakeholders), stakeholder engagement and stakeholder education	[20,45,46,72]
6		Clinical knowledge & health IT	Combining digital means and traditional methods, dentify appropriate technology for patient and adjust use of digital health as needed	[20,42,44-46,63,73]
7		Communication & health IT	Types and methods of communication, digital communication, verbal and non-verbal communication, sharing knowledge	[42,66,67,70]
8	Data protection and security	Cybersecurity	Protection of systems, networks and programmes against digital attacks	[42,45,46]
9	Biostatistics/statistics	Data analytics	Statistical methods and procedures for analysis and interpretation of data and trends	[17,20,42,45,49,64,74,76]
10	Biostatistics/statistics	Data literacy	Collect, manage, evaluate and apply data in a critical manner	[17,20,42,45,66,69,70,72,74-77]
11	Data protection and security	Data protection and privacy	Legal requirements, confidentiality and protection of patient data, as well as identification/authentication; patient's informed consent for therapy and use digital health technologies	[42,45,46,49,66,68-71,73]
12	Decision support	Decision support and decision making	Use, design and limitations of decision support systems for specific problems, evidence-based practice, knowledge management, clinical decision making support	[20,42,45,49,72]
13	eHealth, telematics, telehealth (incl. Interoperability)	eHealth and Electronic Health Records	eHealth applications; health record structure, design, and analysis principles	[42,45,46,49]
14	Ethics and IT	Ethics and legal issues	Confidentiality and its importance (physiological, sociological, psychological, emotional, environmental, cultural, spiritual perspectives and its assessment); ethical codes and practice standards; knowledge of legal, ethical principles and values of the discipline	[42,45,46,67-71,73,78]
15	Financial management	Financial management	Preparation, analysis and evaluation of budget plans, accounting practices, cost-benefit analyses, financial projections	[42,49,73]
16		Health equity & health IT	Social determinants for health, non-medical factors influencing health outcomes, adressing specific needs and reducing disparities among diverse populations (e.g. cultural beliefs, language barriers)	[17,42,45]
17		Healthcare system, economics and regulations	Different types of national and international healthcare delivery models (e.g., hospitals, clinics, ambulatory centres and community health centres, regional health authorities) and their interrelationships	[17,42,45,46]
18	Human Resource Management	Human Resource Management	Management of human resources incl. staff selection, personnel control, laws, staff development	
19	Image and biosignal processing	Image and biosignal processing	Biomedical imaging like visualization methods (e.g., wound monitoring)	[45]
20	Information and communication systems for nursing	Information and communication systems for nursing	Access, disclosure and exchange of data, application design and planning, human-computer interaction, support, architectures and the identification of advantages and limitations of information technology in health care	[49,71,74,76]
21	Information and knowledge management in patient care	Information and knowledge management in patient care	Effective use of data, information and knowledge for scientific research, problem solving and decision making (access to medical knowledge, evaluation, assessment, study design, quantitative and qualitative methods)	[17,42,45,46,48,49,69,71,77,78]
22	Information management in research	Information management in research	Construction and use of clinical register, research methods, literature retrieval and analysis	[42,45,46,48,49,67,68,74,76,78]
23	Information management in teaching, training and education	Information management in teaching, training and education	Tools and methods (teaching and learning systems) to support education and training, informatics to support research and improve information literacy	[45,46,49]
24		Innovation and creativity & health IT	Adoption and integration of innovative technologies, sharing innovative ideas, develop creative soluations; apply new ideas, products, processes, and procedures to individual work role or unit without fear of change	[42,44,70]
25		Interoperability	Health information standards and their appropriate use (e.g., classifications, vocabularies, nomenclature, etc.), information exchange between settings and healthcare providers	[45,46,49,72]
26	IT risk management	IT risk management	Development and maintenance of a risk management system, crisis management strategies, backup platforms and communcation channels, methods for maintaining patient contact and critical communication, proactive crisis response planning	[42,45,67]
27	Nursing documentation (incl. Terminologies)	Nursing documentation (incl. Terminologies)	Objectives, structure and legal basis of medical/nursing documentation, electronic documentation and data management	[42,45,46,49,72]
28		Patient education and engagement	Education and guidance of patients on self-management-techniques, evaluate patient´s digital capabilities	[42,45,63,65,70]
29		Patient safety	Safety protocols, guidelines, patient safety culture, reporting and learning from errors, incident reporting systems, monitoring patient outcomes	[42,45,66,73]
30	Principles of management	Principles of management	Management and organization, purchasing and contract design, structure of health systems, basics of health economics	
31	Principles of nursing informatics	Principles of nursing informatics	Data, information, knowledge, semantics, syntax, software, hardware, networks, HIS	[46,62,74]
32		Problem solving & health IT	Dealing with digital problems, solving problems through digital means	[44,67,70]
33	Process management	Process management	Process optimization, development of new processes and possibly process modeling; analyze existing health processes and identify ways that health data and health information systems can enable improved outcomes	[45,49,64,65]
34		Professional development & health IT	Lifelong learning, commitment to personal growth and self-development	[17,42,44,45,49,63,65-67,69,70]
35	Project management	Project management	Project planning, monitoring, governing, finishing	[42,45,46,49,70,72,74]
36	Quality assurance and quality management	Quality assurance and quality management	Quality analyses, organization and transformation of data into reliable and consistent information for different target groups; Understanding of various quality improvement models; tools including quality planning and assurance and control	[42,45,46,67]
37	Ressource planning and logistics	Ressource planning and logistics	Management of medical supplies, drugs, instruments as well as logistics concepts and their implementation	[45]
38	Assistive technologies	Smart assistive technologies	Ubiquitous technologies (e.g., in the context of assisted living)	
39		Social and behavioural science & health IT	Effects of social, behavioral, and psychological models applicable to health informatics from multiple levels including individuals, organizations, social groups, and society.	[17,68,69]
40		Sociotechnical aspects	Foundational knowledge, skills, and attitudes to identify social behavioral models and human factors engineering to better understand the interaction between users and information technologies, and to be knowledgeable of information system design	[17,45]
41	Strategic management and leadership	Strategic management, leadership and intrapreneurship	Management of information as a strategic resource and knowledge about modern management styles and methods, professionalism and education, stewardship	[17,20,42,45,49,64,67,69,74]
42		Teamwork and collaboration & health IT	Tools such as online platforms for collaboration, document-sharing systems; roles/responsibilities for collaborative practice, interprofessional teamwork and teambased practice	[17,42,45,66,67,70,73]
43	eHealth, telematics, telehealth (incl. Interoperability)	telehealth and mHealth	Technologies, platforms, apps, and equipment used in virtual healthcare delivery, such as videoconferencing tools; consumer health informatics	[42,45,46,49]

Appendix C

Mapping of Core Competency Areas with IMIA Domains in Biomedical and Health Informatics

BMHI Core Principles and Specialization	Computer, Data and Information Science	Health Sciences and Services	Social and Behavioural Sciences	Management Sciences
Nursing documentation incl. Terminologies	Applied Computer Science	patient education and engagement	Change and stakeholder management	financial management
information management in teaching, raining and education	Artificial Intelligence	patient safety	Communication & health IT	human resource management
decision support and decision making	Cybersecurity	quality assurance and quality management	Data protection and privacy	principles of management
information management in research	Data analytics	healthcare system, economics and regulations	health equity & health IT	process management
telehealth and mHealth	IT risk management		Innovation and creativity & health IT	professional management & health IT
smart assistive technologies			problem solving & health IT	project management
principles of nursing informatics			social and behavioural sciences & health IT	resource planning an logistics
eHealth and electronic health records			sociotechnical aspects	strategic management, leadership and intrapreneuship
information and communication systems for nursing			teamwork and collaboration & health IT	
Interoperability			ethics & health IT	
Basic computer literacy / digital literacy				
information and knowledge management in patient care				
Image and biosignal processing				
Care coordination and integration & health IT				
clinical knowledge & health IT				
Data literacy				

Appendix D

Case study for the concrete application of the recommentations

Please specify the area from which your case study originates:

☐ university study program (please select: ☐ bachelor ☐ master ☐ further education) ☐ vocational training ☐ general further education ☐ specialist further education ☐ other: [please indicate]

Competence area (multiple selection of different competence areas are addressed on one course)	Course characteristics (e. g. extent, credit points)	Target group	Learning method (e. g. in presence/online/hybrid, problem based/case based learning)	Learning objektives and assessment	Description of course content
☐ Applied computer science<br>☐ Artificial Intelligence<br>☐ Basic computer literacy / digital literacy<br>☐ Care coordination and integration & health IT<br>☐ Change and stakeholder management<br>☐ Clinical knowledge & health IT<br>☐ Communication & health IT<br>☐ Cybersecurity<br>☐ Data analytics<br>☐ Data literacy<br>☐ Data protection and privacy<br>☐ Decision support und decision making<br>☐ eHealth and electronic health records<br>☐ Ethics and health IT<br>☐ Financial management<br>☐ Health equity & health IT<br>☐ Healthcare system, economics and regulations<br>☐ Human resource management<br>☐ Image and biosignal processing<br>☐ Information and communication systems for nursing<br>☐ Information and knowledge management in patient care<br>☐ Information management in research<br>☐ Information management in teaching, training and education<br>☐ Innovation and creativity & health IT<br>☐ Interoperability<br>☐ IT risk management<br>☐ Nursing documentation (incl. terminologies)<br>☐ Patient education and engagement<br>☐ Patient safety<br>☐ Principles of management<br>☐ Principles of nursing informatics<br>☐ Problem solving & health IT<br>☐ Process management<br>☐ Professional management & health IT<br>☐ Project management<br>☐ Quality assurance and quality management<br>☐ Resource planning and logistics<br>☐ Smart assistive technologies<br>☐ Social and behavioural sciences & health IT<br>☐ Sociotechnical aspects<br>☐ Strategic management, leadership and intrapreneurship<br>☐ Teamwork and collaboration & health IT<br>☐ Telehealth and mHealth					

Appendix E

Relevance Ratings for Core Competency Areas and All Roles

Clinical Nursing (all countries and professions)

No.	Competence Area	Relevance Rating	No.	Competence Area	Relevance Rating
1	Patient safety [n=112]	88,1	23	Problem solving & health IT [n=112]	63,7
2	Nursing documentation [n=112]	86,8	24	Innovation and creativity & health IT [n=112]	63,3
3	Basic computer literacy / digital literacy [n=117]	86,0	25	Change and stakeholder management [n=114]	62,6
4	Ethics and legal issues [n=113]	83,9	26	Interoperability [n=112]	62,6
5	Data protection and privacy [n=113]	83,6	27	Information management in teaching, training and education [n=112]	62,2
6	Patient education and engagement [n=112]	82,9	28	Social and behavioural science & health IT [n=112]	61,4
7	Communication & health IT [n=113]	82,8	29	Sociotechnical aspects [n=112]	61,4
8	Clinical knowledge & health IT [n=113]	81,7	30	Artifical Intelligence [n=117]	59,8
9	eHealth and electronic health records [n=113]	80,2	31	Data analytics [n=113]	59,4
10	Care coordination and integration & health IT [n=115]	79,8	32	IT risk management [n=112]	59,2
11	Information and knowledge management in patient care [n=113]	78,6	33	Process management [n=112]	58,6
12	Decision support and decision making [n=113]	76,6	34	Healthcare system, economics and regulations [n=113]	58,5
13	Telehealth and mHealth [n=112]	75,7	35	Information management in research [n=113]	57,1
14	Teamwork and collaboration & health IT [n=112]	74,7	36	Ressource planning and logistics [n=112]	56,2
15	Cybersecurity [n=113]	74,5	37	Project management [n=112]	56,0
16	Information and communication systems for nursing [n=113]	72,7	38	Image and biosignal processing [n=113]	54,1
17	Health equity & health IT [n=113]	72,5	39	Strategic management, leadership and intrapreneurship [n=112]	53,0
18	Data literacy [n=113]	72,0	40	Principles of management [n=112]	53,0
19	Principles of nursing informatics [n=112]	71,6	41	Human resource management [n=112]	49,9
20	Professional development & health IT [n=112]	71,6	42	Financial management [n=112]	47,1
21	Quality assurance and quality management [n=112]	66,7	43	Applied computer science [n=117]	46,0
22	Smart assistive technologies [n=112]	66,2			

Nursing Management (all countries and professions, n=81)

No.	Competence Area	Relevance Rating	No.	Competence Area	Relevance Rating
1	Basic computer literacy / digital literacy	89,3	23	Information and communication systems for nursing	80,5
2	Ethics and legal issues	88,9	24	Decision support and decision making	79,6
3	Principles of management	88,5	25	Principles of nursing informatics	79,6
4	Data protection and privacy	88,2	26	Data analytics	79,4
5	Patient safety	87,8	27	Health equity & health IT	79,4
6	Care coordination and integration & health IT	87,0	28	Healthcare system, economics and regulations	78,6
7	Nursing documentation	85,7	29	Clinical knowledge & health IT	78,5
8	Change and stakeholder management	85,4	30	Patient education and engagement	77,3
9	Strategic management, leadership and intrapreneurship	84,9	31	Telehealth and mHealth	76,1
10	Human resource management	84,8	32	IT risk management	75,9
11	Cybersecurity	84,8	33	Innovation and creativity & health IT	73,8
12	Teamwork and collaboration & health IT	84,8	34	Problem solving & health IT	73,8
13	Data literacy	84,4	35	Interoperability	73,7
14	Quality assurance and quality management	84,0	36	Information management in teaching, training and education	73,1
15	Ressource planning and logistics	83,8	37	Information management in research	69,9
16	eHealth and electronic health records	83,7	38	Social and behavioural science & health IT	69,6
17	Project management	83,3	39	Sociotechnical aspects	69,6
18	Information and knowledge management in patient care	83,1	40	Artifical Intelligence [n=82]	68,9
19	Process management	83,0	41	Smart assistive technologies	67,9
20	Communication & health IT	82,8	42	Applied computer science	54,4
21	Professional development & health IT	82,6	43	Image and biosignal processing	52,6
22	Financial management	82,1			

Quality Management (all countries and professions, n=63)

No.	Competence Area	Relevance Rating	No.	Competence Area	Relevance Rating
1	Quality assurance and quality management	91,5	23	Care coordination and integration & health IT	75,5
2	Patient safety	90,0	24	Professional development & health IT	74,4
3	Ethics and legal issues	89,0	25	Interoperability	73,3
4	Basic computer literacy / digital literacy	85,7	26	Healthcare system, economics and regulations	72,9
5	Data literacy	85,7	27	IT risk management	72,6
6	Data protection and privacy	85,7	28	Innovation and creativity & health IT	72,5
7	Data analytics	83,9	29	Problem solving & health IT	72,0
8	Nursing documentation	83,6	30	Information management in teaching, training and education	71,7
9	Information and knowledge management in patient care	82,9	31	Patient education and engagement	71,3
10	Process management	82,0	32	Ressource planning and logistics	69,4
11	eHealth and electronic health records	81,6	33	Sociotechnical aspects	69,0
12	Project management	79,1	34	Strategic management, leadership and intrapreneurship	68,4
13	Communication & health IT	78,4	35	Telehealth and mHealth	68,3
14	Decision support and decision making	78,4	36	Artifical Intelligence	68,1
15	Cybersecurity	78,3	37	Social and behavioural science & health IT	67,7
16	Information and communication systems for nursing	78,3	38	Principles of management	67,5
17	Teamwork and collaboration & health IT	78,1	39	Financial management	64,2
18	Clinical knowledge & health IT	77,8	40	Smart assistive technologies	61,5
19	Change and stakeholder management	77,0	41	Applied computer science	59,9
20	Information management in research	76,5	42	Human resource management	59,9
21	Health equity & health IT	75,9	43	Image and biosignal processing	56,5
22	Principles of nursing informatics	75,7			

Coordination of Interprofessional Care (all countries and professions)

No.	Competence Area	Relevance Rating	No.	Competence Area	Relevance Rating
1	Care coordination and integration & health IT [n=62]	87,6	23	Interoperability [n=61]	70,7
2	Ethics and legal issues [n=62]	86,0	24	Quality assurance and quality management [n=62]	70,2
3	Basic computer literacy / digital literacy [n=62]	84,8	25	Project management [n=62]	68,9
4	Patient safety [n=62]	84,7	26	Principles of nursing informatics [n=62]	68,6
5	Teamwork and collaboration & health IT [n=62]	83,6	27	IT risk management [n=62]	66,9
6	Communication & health IT [n=63]	83,3	28	Innovation and creativity & health IT [n=62]	66,8
7	Nursing documentation [n=62]	82,4	29	Problem solving & health IT [n=62]	66,1
8	Data protection and privacy [n=61]	81,6	30	Data analytics [n=62]	66,0
9	Information and knowledge management in patient care [n=62]	80,3	31	Information management in teaching, training and education [n=61]	65,7
10	Information and communication systems for nursing [n=62]	79,5	32	Ressource planning and logistics [n=62]	65,3
11	eHealth and electronic health records [n=62]	79,2	33	Principles of management [n=62]	64,8
12	Clinical knowledge & health IT [n=62]	79,0	34	Smart assistive technologies [n=62]	64,7
13	Cybersecurity [n=62]	78,9	35	Information management in research [n=62]	63,6
14	Data literacy [n=62]	75,5	36	Artifical Intelligence [n=62]	63,4
15	Patient education and engagement [n=62]	74,5	37	Strategic management, leadership and intrapreneurship [n=62]	63,4
16	Decision support and decision making [n=62]	73,9	38	Social and behavioural science & health IT [n=62]	62,6
17	Process management [n=61]	73,8	39	Sociotechnical aspects [n=62]	62,4
18	Telehealth and mHealth [n=62]	73,4	40	Human resource management [n=62]	56,0
19	Professional development & health IT [n=62]	73,2	41	Financial management [n=62]	55,0
20	Health equity & health IT [n=61]	72,8	42	Applied computer science [n=62]	54,4
21	Change and stakeholder management [n=62]	72,6	43	Image and biosignal processing [n=61]	51,3
22	Healthcare system, economics and regulations [n=61]	71,5			

IT Management in Nursing (all countries and professions, n=64)

No.	Competence Area	Relevance Rating	No.	Competence Area	Relevance Rating
1	Basic computer literacy / digital literacy	90,6	23	Data analytics	81,1
2	Data protection and privacy	89,5	24	Decision support and decision making	80,9
3	Cybersecurity	89,2	25	Change and stakeholder management	80,8
4	eHealth and electronic health records	88,9	26	Nursing documentation	80,6
5	Principles of nursing informatics	88,0	27	Information and knowledge management in patient care	78,6
6	Problem solving & health IT	87,8	28	Health equity & health IT	78,1
7	Communication & health IT	87,7	29	Quality assurance and quality management	78,0
8	IT risk management	86,7	30	Patient safety	77,5
9	Teamwork and collaboration & health IT	85,3	31	Healthcare system, economics and regulations	76,6
10	Data literacy	85,2	32	Principles of management	75,0
11	Ethics and legal issues	84,7	33	Strategic management, leadership and intrapreneurship	74,5
12	Information and communication systems for nursing	84,7	34	Information management in research	72,8
13	Interoperability	84,7	35	Smart assistive technologies	72,8
14	Innovation and creativity & health IT	84,4	36	Ressource planning and logistics	72,2
15	Artifical Intelligence	84,2	37	Sociotechnical aspects	72,2
16	Process management	83,9	38	Information management in teaching, training and education	71,3
17	Telehealth and mHealth	82,8	39	Social and behavioural science & health IT	70,9
18	Project management	82,5	40	Financial management	67,8
19	Care coordination and integration & health IT	82,3	41	Human resource management	66,4
20	Clinical knowledge & health IT	82,3	42	Image and biosignal processing	62,8
21	Professional development & health IT	82,2	43	Patient education and engagement	62,8
22	Applied computer science	82,0			

Appendix F

Changes in Relevance Ratings between 2018 and 2026 for All Core Competence Areas and All Roles

Clinical Nursing

2026			2018		
Rank	Core Competence Area	Rating	Rating	Rank	Core Competence Area
1	Patient safety	88.1			
2	Nursing documentation	86.8	94.4	1	Nursing documentation
3	Basic computer literacy / digital literacy	86.0			
4	Ethics and legal issues	83.9	79.5	5	Ethics and IT
5	Data protection and privacy	83.6	80.0	4	Data protection and security
6	Patient education and engagement	82.9			
7	Communication & health IT	82.8			
8	Clinical knowledge & health IT	81.7			
9	eHealth and electronic health records	80.2	69.5	9	eHealth, telematics and telehealth
10	Care coordination and integration & health IT	79.8			
11	Information and knowledge management in patient care	78.6	82.2	2	Information and knowledge management
12	Decision support and decision making [n=113]	76.6	70.2	8	Decision support by IT
13	Telehealth and mHealth	75.7			
14	Teamwork and collaboration & health IT	74.7			
15	Cybersecurity	74.5			
16	Information and communication systems for nursing	72.7	75.1	6	Information systems and communication systems
17	Health equity & health IT	72.5			
18	Data literacy	72.0			
19	Principles of nursing informatics	71.6	80.5	3	Principles of nursing informatics
20	Professional development & health IT	71.6			
21	Quality assurance and quality management	66.7	72.0	7	Quality management
22	Smart assistive technologies	66.2	69.0	10	Assistive technology for ageing people
23	Problem solving & health IT	63.7			
24	Innovation and creativity & health IT	63.3			
25	Change and stakeholder management	62.6	58.0	15	Change management and stakeholder management
26	Interoperability	62.6			
27	Information management in teaching, training and education	62.2	61.7	12	Information management in teaching, training and education
28	Social and behavioural science & health IT	61.4			
29	Sociotechnical aspects	61.4			
30	Artifical Intelligence	59.8			
31	Data analytics	59.4	47.8	23	Biostatistics/statistics
32	IT risk management	59.2	61.2	13	IT risk management
33	Process management	58.6	67.8	11	Process management
34	Healthcare system, economics and regulations	58.5			
35	Information management in research [n=113]	57.1	51.0	22	Information management in research
36	Ressource planning and logistics	56.2	56.6	18	Ressource planning and logistics
37	Project management	56.0	55.6	19	Project management
38	Image and biosignal processing	54.1	55.6	20	Biomedical imaging and signal processing
39	Strategic management, leadership and intrapreneurship	53.0	57.1	16	Strategic management and leadership
40	Principles of management	53.0	59.8	14	Principles of management
41	Human resource	49.9	57.1	17	Human resource management
42	Financial management	47.1	47.6	24	Financial management
43	Applied computer science	46.0	53.7	21	Applied computer science/informatics

Higher relevance rating in 2026 than in 2018 (at least 5 points) (green)
Lower relevance rating in 2026 than in 2018 (at least 5 points) (orange)

Nursing Management

2026			2018		
Rank	Core Competence Area	Rating	Rating	Rank	Core Competence Area
1	Basic computer literacy / digital literacy	90.3			
2	Ethics and legal issues	88.7	80.5	10	Ethics and IT
3	Principles of management	87.6	87.9	2	Principles of management
4	Data protection and privacy	87.6	80.2	11	Data protection and security
5	Patient safety	87.5			
6	Care coordination and integration & health IT	86.7			
7	Nursing documentation	86.5	92.1	1	Nursing documentation
8	Change and stakeholder management	86.4	84.2	6	Change management and stakeholder management
9	Strategic management, leadership and intrapreneurship	86.0	86.7	3	Strategic management and leadership
10	Human resource management	85.4	84.4	5	Human resource management
11	Cybersecurity	85.1			
12	Teamwork and collaboration & health IT	84.6			
13	Data literacy	84.4			
14	Quality assurance and quality management	84.4	85.1	4	Quality management
15	Ressource planning and logistics	84.3	76.0	13	Ressource planning and logistics
16	eHealth and electronic health records	83.8	66.3	19	eHealth, telematics and telehealth
17	Project management	83.5	76.3	12	Project management
18	Information and knowledge management in patient care	83.5	84.0	7	Information and knowledge management
19	Process management	83.5	81.2	9	Process management
20	Communication & health IT	82.5			
21	Professional development & health IT	82.1			
22	Financial management	81.0	73.7	16	Financial management
23	Information and communication systems for nursing	80.6	75.1	14	Information systems and communication systems
24	Decision support and decision making	79.8	74.7	15	Decision support by IT
25	Principles of nursing informatics	79.5	82.3	8	Principles of nursing informatics
26	Data analytics	79.2	59.5	22	Biostatistics/statistics
27	Health equity & health IT	78.9			
28	Healthcare system, economics and regulations	78.1			
29	Clinical knowledge & health IT	77.8			
30	Patient education and engagement	75.4			
31	Telehealth and mHealth	74.9			
32	IT risk management	74.6	73.3	17	IT risk management
33	Innovation and creativity & health IT	74.1			
34	Problem solving & health IT	74.0			
35	Interoperability	73.8			
36	Information management in teaching, training and education	72.5	70.0	18	Information management in teaching, training and education
37	Information management in research	71.3	63.3	20	Information management in research
38	Social and behavioural science & health IT	70.0			
39	Sociotechnical aspects	69.4			
40	Artifical Intelligence [n=82]	68.6			
41	Smart assistive technologies	67.8	63.3	21	Assistive technology for ageing people
42	Applied computer science	56.2	57.4	23	Applied computer science/informatics
43	Image and biosignal processing	52.2	54.7	24	Biomedical imaging and signal processing

Quality Management

2026			2018		
Rank	Core Competence Area	Rating	Rating	Rank	Core Competence Area
1	Quality assurance and quality management	91.5	96.1	1	Quality Management
2	Patient safety	90.0			
3	Ethics and legal issues	89.0	75.9	13	Ethics and IT
4	Basic computer literacy / digital literacy	85.7			
5	Data literacy	85.7			
6	Data protection and privacy	85.7	79.5	7	Data protection and security
7	Data analytics	83.9	76.6	12	Biostatistics/statistics
8	Nursing documentation	83.6	84.4	3	Nursing documentation
9	Information and knowledge management in patient care	82.9	83.2	4	Information and knowledge management
10	Process management	82.0	86.8	2	Process management
11	eHealth and electronic health records	81.6	69.8	17	eHealth, telematics and telehealth
12	Project management	79.1	78.5	8	Project management
13	Communication & health IT	78.4			
14	Decision support and decision making	78.4	72.7	15	Decision support by IT
15	Cybersecurity	78.3			
16	Information and communication systems for nursing	78.3	82.0	5	Information systems and communication systems
17	Teamwork and collaboration & health IT	78.1			
18	Clinical knowledge & health IT	77.8			
19	Change and stakeholder management	77.0	77.6	10	Change management and stakeholder management
20	Information management in research	76.5	72.4	16	Information management in research
21	Health equity & health IT	75.9			
22	Principles of nursing informatics	75.7	80.2	6	Principles of nursing informatics
23	Care coordination and integration & health IT	75.5			
24	Professional development & health IT	74.4			
25	Interoperability	73.3			
26	Healthcare system, economics and regulations	72.9			
27	IT risk management	72.6	73.9	14	IT risk management
28	Innovation and creativity & health IT	72.5			
29	Problem solving & health IT	72.0			
30	Information management in teaching, training and education	71.7	67.1	19	Information management in teaching, training and education
31	Patient education and engagement	71.3			
32	Ressource planning and logistics	69.4	65.4	21	Ressource planning and logistics
33	Sociotechnical aspects	69.0			
34	Strategic management, leadership and intrapreneurship	68.4	77.1	11	Strategic management and leadership
35	Telehealth and mHealth	68.3			
36	Artifical Intelligence [n=70]	68.1			
37	Social and behavioural science & health IT	67.7			
38	Principles of management	67.5	78.5	9	Principles of management
39	Financial management	64.2	65.4	20	Financial management
40	Smart assistive technologies	61.5	54.9	23	Assistive technology for ageing people
41	Applied computer science [n=70]	59.9	63.7	22	Applied computer science/informatics
42	Human resource management	59.9	68.8	18	Human resource management
43	Image and biosignal processing	56.5	49.5	24	Biomedical imaging and signal processing

Thieme

Coordination of Inter-Professional Care

2026			2018		
Rank	Core Competence Area	Rating	Rating	Rank	Core Competence Area
1	Care coordination and integration & health IT	87.6			
2	Ethics and legal issues	86.0	78.8	6	Ethics and IT
3	Basic computer literacy / digital literacy	84.8			
4	Patient safety	84.7			
5	Teamwork and collaboration & health IT	83.6			
6	Communication & health IT [n=63]	83.3			
7	Nursing documentation	82.4	83.4	3	Nursing documentation
8	Data protection and privacy [n=61]	81.6	85.9	1	Data protection and security
9	Information and knowledge management in patient care	80.3	85.4	2	Information and knowledge management
10	Information and communication systems for nursing	79.5	81.5	5	Information systems and communication systems
11	eHealth and electronic health records	79.2	77.6	7	eHealth, telematics and telehealth
12	Clinical knowledge & health IT	79.0			
13	Cybersecurity	78.9			
14	Data literacy	75.5			
15	Patient education and engagement	74.5			
16	Decision support and decision making	73.9	70.0	16	Decision support by IT
17	Process management [n=61]	73.8	83.2	4	Process management
18	Telehealth and mHealth	73.4			
19	Professional development & health IT	73.2			
20	Health equity & health IT [n=61]	72.8			
21	Change and stakeholder management	72.6	73.7	11	Change management and stakeholder management
22	Healthcare system, economics and regulations [n=61]	71.5			
23	Interoperability [n=61]	70.7			
24	Quality assurance and quality management	70.2	77.1	8	Quality Management
25	Project management	68.9	72.4	13	Project management
26	Principles of nursing informatics	68.6	74.6	9	Principles of nursing informatics
27	IT risk management	66.9	67.8	18	IT risk management
28	Innovation and creativity & health IT	66.8			
29	Problem solving & health IT	66.1			
30	Data analytics	66.0	55.6	23	Biostatistics/statistics
31	Information management in teaching, training and education [r	65.7	66.1	19	Information management in teaching, training and education
32	Ressource planning and logistics	65.3	71.7	14	Ressource planning and logistics
33	Principles of management	64.8	74.6	10	Principles of management
34	Smart assistive technologies	64.7	70.2	15	Assistive technology for ageing people
35	Information management in research	63.6	60.5	22	Information management in research
36	Artifical Intelligence	63.4			
37	Strategic management, leadership and intrapreneurship	63.4	72.7	12	Strategic management and leadership
38	Social and behavioural science & health IT	62.6			
39	Sociotechnical aspects	62.4			
40	Human resource management	56.0	68.0	17	Human resource management
41	Financial management	55.0	62.0	21	Financial management
42	Applied computer science	54.4	64.9	20	Applied computer science/informatics
43	Image and biosignal processing [n=61]	51.3	55.4	24	Biomedical imaging and signal processing

IT-Management in Nursing

2026			2018		
**Rank**	**Core Competence Area**	**Rating**	**Rating**	**Rank**	**Core Competence Area**
1	Basic computer literacy / digital literacy	90.6			
2	Data protection and privacy	89.5	89.0	3	Data protection and security
3	Cybersecurity	89.2			
4	eHealth and electronic health records	88.9	80.0	14	eHealth, telematics and telehealth
5	Principles of nursing informatics	88.0	89.5	2	Principles of nursing informatics
6	Problem solving & health IT	87.8			
7	Communication & health IT	87.7			
8	IT risk management	86.7	86.8	4	IT risk management
9	Teamwork and collaboration & health IT	85.3			
10	Data literacy	85.2			
11	Ethics and legal issues	84.7	83.4	11	Ethics and IT
12	Information and communication systems for nursing	84.7	89.5	1	Information systems and communication systems
13	Interoperability	84.7			
14	Innovation and creativity & health IT	84.4			
15	Artifical Intelligence	84.2			
16	Process management	83.9	86.1	6	Process management
17	Telehealth and mHealth	82.8			
18	Project management	82.5	86.8	5	Project management
19	Care coordination and integration & health IT	82.3			
20	Clinical knowledge & health IT	82.3			
21	Professional development & health IT	82.2			
22	Applied computer science	82.0	83.4	9	Applied computer science/informatics
23	Data analytics	81.1	67.3	22	Biostatistics/statistics
24	Decision support and decision making	80.9	85.4	8	Decision support by IT
25	Change and stakeholder management	80.8	80.5	13	Change management and stakeholder management
26	Nursing documentation	80.6	83.4	10	Nursing documentation
27	Information and knowledge management in patient care	78.6	86.1	7	Information and knowledge management
28	Health equity & health IT	78.1			
29	Quality assurance and quality management	78.0	80.7	12	Quality Management
30	Patient safety	77.5			
31	Healthcare system, economics and regulations	76.6			
32	Principles of management	75.0	79.3	16	Principles of management
33	Strategic management, leadership and intrapreneurship	74.5	79.5	15	Strategic management and leadership
34	Information management in research	72.8	71.5	18	Information management in research
35	Smart assistive technologies	72.8	70.2	19	Assistive technology for ageing people
36	Ressource planning and logistics	72.2	71.7	17	Ressource planning and logistics
37	Sociotechnical aspects	72.2			
38	Information management in teaching, training and education	71.3	74.4	16	Information management in teaching, training and education
39	Social and behavioural science & health IT	70.9			
40	Financial management	67.8	69.8	20	Financial management
41	Human resource management	66.4	69.8	21	Human resource management
42	Image and biosignal processing	62.8	62.4	24	Biomedical imaging and signal processing
43	Patient education and engagement	62.8			
